# Genome-Wide Association Mapping and Gene Expression Analysis Reveal the Negative Role of *OsMYB21* in Regulating Bacterial Blight Resistance in Rice

**DOI:** 10.1186/s12284-021-00501-z

**Published:** 2021-06-29

**Authors:** Wu Yang, Junliang Zhao, Shaohong Zhang, Luo Chen, Tifeng Yang, Jingfang Dong, Hua Fu, Yamei Ma, Lian Zhou, Jian Wang, Wei Liu, Qing Liu, Bin Liu

**Affiliations:** 1 Rice Research Institute, Guangdong Academy of Agricultural Sciences, Guangzhou, 510640 China; 2 Guangdong Key Laboratory of New Technology in Rice Breeding, Guangzhou, 510640 China

**Keywords:** GWAS, Bacterial blight, *OsMYB21*, Rice

## Abstract

**Background:**

Bacterial blight (BB), caused by *Xanthomonas oryzae pv. oryzae* (*Xoo*), is one of the most devastating diseases in rice all over the world. Due to the diversity and rapid evolution of *Xoo*, identification and use of the non-race specific quantitative resistance QTLs has been considered the preferred strategy for effective control of this disease. Although numerous QTLs for BB resistance have been identified, they haven’t been effectively used for improvement of BB resistance in rice due to their small effects and lack of knowledge on the function of genes underlying the QTLs.

**Results:**

In the present study, a genome-wide association study of BB resistance was performed in a rice core collection from South China. A total of 17 QTLs were identified to be associated with BB resistance. Among them, 13 QTLs were newly identified in the present study and the other 4 QTLs were co-localized with the previously reported QTLs or *Xa* genes that confer qualitative resistance to *Xoo* strains. Particularly, the *qBBR11–4* on chromosome 11 explained the largest phenotypic variation in this study and was co-localized with the previously identified QTLs for BB and bacterial leaf streak (BLS) resistance against diverse strains in three studies, suggesting its broad-spectrum resistance and potential value in rice breeding. Through combined analysis of differential expression and annotations of the predicted genes within *qBBR11–4* between two sets of rice accessions selected based on haplotypes and disease phenotypes, we identified the transcription factor *OsMYB21* as the candidate gene for *qBBR11–4*. The *OsMYB21* overexpressing plants exhibited decreased resistance to bacterial blight, accompanied with down-regulation of several defense-related genes compared with the wild-type plants.

**Conclusion:**

The results suggest that *OsMYB21* negatively regulates bacterial blight resistance in rice, and this gene can be a promising target in rice breeding by using the gene editing method. In addition, the potential candidate genes for the 13 novel QTLs for BB resistance were also analyzed in this study, providing a new source for cloning of genes associated with BB resistance and molecular breeding in rice.

**Supplementary Information:**

The online version contains supplementary material available at 10.1186/s12284-021-00501-z.

## Background

Bacterial blight (BB), caused by *Xanthomonas oryzae pv. oryzae* (*Xoo*), is one of the most devastating rice diseases all over the world (Savary et al. 2019). Nowadays, developing and deploying resistance rice cultivars is considered the most economic and environmentally friendly way to control this disease (Zhang et al. 2017). Rice resistance against BB can be generally divided into two main categories, the qualitative resistance controlled by major resistance (*R*) genes, and the quantitative resistance conferred by multiple minor genes or quantitative trait loci (QTLs) (Ramalingam et al. 2003; Deng et al. 2012; Bossa-Castro et al. 2018). So far, over 40 *R* genes that confer qualitative resistance to BB has been identified (Jiang et al. 2020) and 11 of them (*Xa1*, *Xa3/Xa26*, *Xa4*, *xa5*, *Xa10*, *xa13*, *Xa21*, *Xa23*, *xa25*, *Xa27*, *xa41*) have been cloned successfully by using map-based cloning strategy or knowledge-based molecular screening (Yoshimura et al. 1998; Han et al. 2014; Hutin et al. 2015; Wang et al. 2015; Ji et al. 2018). The *R* genes with comparatively broader spectra of resistance, such as *Xa3*, *Xa4, Xa7, xa13, Xa21 and Xa23*, have been widely used in rice breeding programs, and many resistant rice cultivars have been released (Huang et al. 1997; Han et al. 2014; Wang et al. 2015; Zhang et al. 2015; Hu et al. 2017; Jiang et al. 2020). Although the disease resistance conferred by a single *R* gene is usually effective against certain races of the *Xoo* pathogen, the resistance is easily breakdown due to a greater selection pressure on pathogen evolution. Conversely, the quantitative resistance mediated by QTLs is presumably non-race specific and is considered to be more durable (Liu et al. 2016). Thus, it has attracted more attention in the past decades and more than 70 QTLs for BB resistance have been identified (Li et al. 2006; Han et al. 2014; Djedatin et al. 2016; Dilla-Ermita et al. 2017; Zhang et al. 2017; Bossa-Castro et al. 2018). Despite quantitative resistance has been considered as a preferred strategy to achieve durable resistance and numerous QTLs for BB resistance have been identified, marker-assisted selection has not been effectively used for improvement of BB resistance in rice. This issue is attributed to the polygenic nature of the trait and each QTL has small effect. It is difficult to accumulate multiple QTLs with small effects in breeding. In addition, most of the QTLs for BB resistance were identified and mapped using bi-parental population QTL analysis in the past decades. Because of limited molecular markers used and less recombinants in a primary mapping population, most of the QTLs for BB resistance are mapped to a region of 10 ~ 30 cM. Since a prerequisite for successful marker-assisted selection (MAS) is the availability of the markers that closely linked with the target gene, the inaccuracy of QTL mapping hinders the application of MAS. Therefore, discovery of the large-effect QTLs and use of a more powerful approach for genetic dissection of complex traits are crucial to address this issue.

Encouragingly, the rapid development of high through-put sequencing technology, SNP array technology and their applications for genotyping lead to the development of genome-wide association study (GWAS). Compared with conventional bi-parental population QTL analysis, GWAS has two main advantages: (1) it can use nature population instead of bi-parental population. The rice varieties used in GWAS contain much more genetic diversity than the bi-parental lines used in segregation populations. Because of using diverse germplasm for QTL mapping in GWAS, it favors the identification of large-effect and novel QTLs; (2) most GWAS can result in a relatively high mapping resolution due to the existence of numerous historical recombination events (Takeda and Matsuoka 2008) and using plenty of SNPs for association mapping. Therefore, GWAS provides a powerful tool for large-scale and precise identification of QTLs for the complex traits like BB resistance in germplasm (Zhao et al. 2011; Han and Huang 2013; Zhang et al. 2017; Zhai et al. 2018).

To search for large-effect and novel BB resistant QTLs and their causal genes, GWAS of BB resistance was conducted using a diverse core panel consisting of 255 landraces and 58 modern varieties from South China which have been sequenced in the present study. We identified 17 QTLs for BB resistance, including 13 novel QTLs and 4 known QTLs/genes identified in the previous studies, implying the reliability of the GWAS results and diversity of the rice germplasm used in this study. Among the 17 QTLs for BB resistance, *qBBR11–4* explained the largest diseased phenotypic variation. Through gene differential expression analysis, annotations of the predicted genes within *qBBR11–4* and transgenic analysis, the MYB transcription factor 21 (*OsMYB21*), *Os11g0684000*, was validated as the functional target genes underlying the QTL *qBBR11–4*. The *OsMYB21* overexpressing plants showed significantly increased susceptibility to bacterial blight. Moreover, the expression levels of several pathogenesis-related (*PR*) genes were remarkably down-regulated in the transgenic plants compared to the wild-type plants. The results from this study provides good sources for discovery of new genes related to bacterial blight resistance, and *OsMYB21* can be a promising target in rice breeding by using the gene editing method.

## Results

### The Phylogenetic Analysis of the Core Collection and their Reactions to Bacterial Blight

**Fig. 1:**
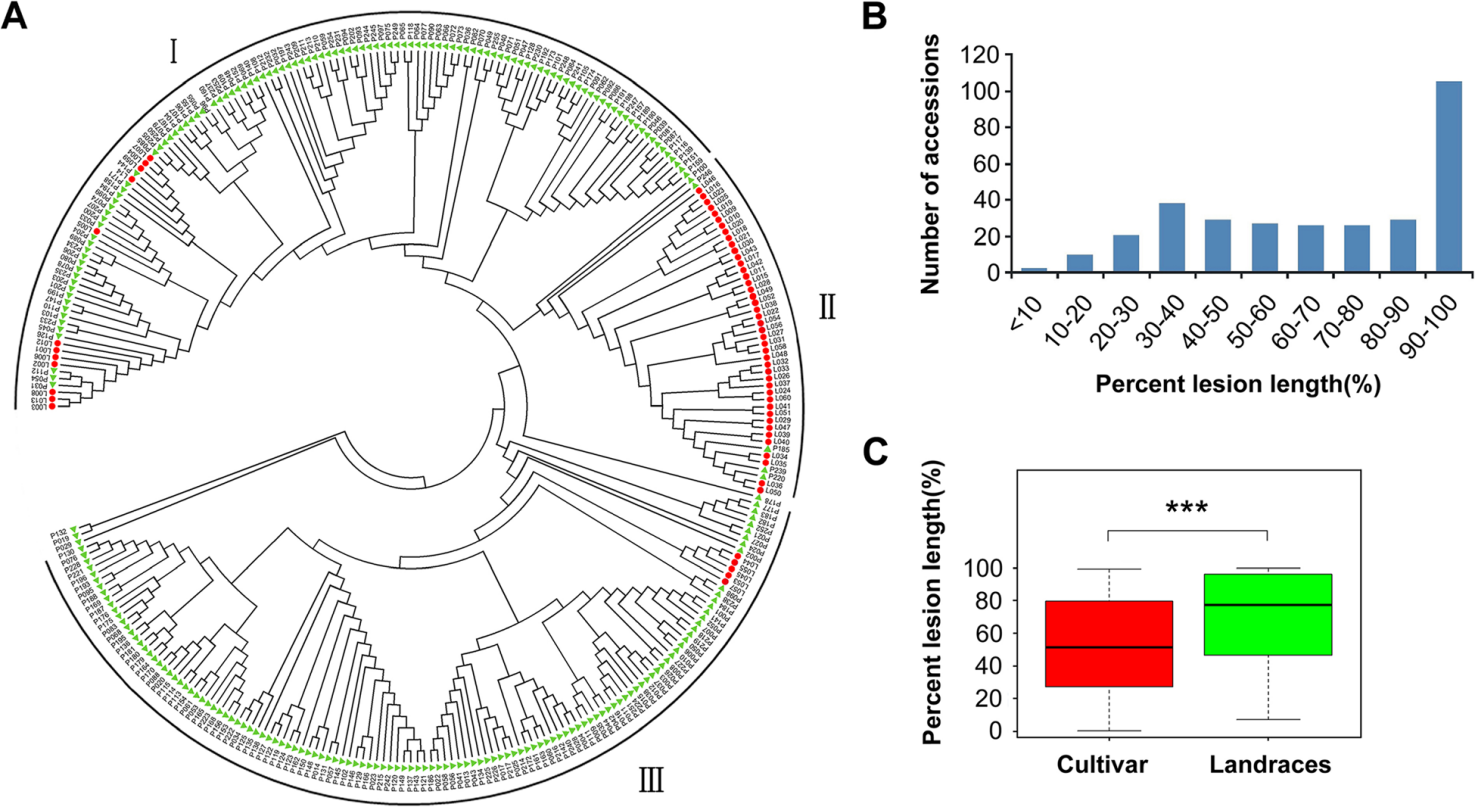
The phylogenetic tree and phenotypic distribution of 313 rice accessions used in this study. **A** The phylogenetic tree of 313 rice accessions. The round (red) and trigonal (green) shape represent modern cultivar and landraces, respectively. **B** Phenotypic distribution of 313 rice accessions. **C** Comparison of the average percent lesion length between modern cultivars and landraces. Asterisk indicates significant difference (Student’s *t*-test, ***P < 0.001)

The 313 rice accessions used in this study are the core collection of the rice germplasms collected by Guangdong Academy of Agricultural Science, China. This core collection was selected from grouping of the original germplasm based on their 26 traits and genotypes determined by 384 SNPs, and represents 90% of the diversity of the original collection (unpublished data). The core collection panel consists of 255 landraces and 58 modern cultivars from South China (Table S1). All the accessions were genotyped using next-generation sequencing technology and 643,841 SNPs were selected for phylogenetic and GWAS analysis in the present study. The population structure of the panel was shown by phylogenetic tree (Fig. 1A). According to the phylogenetic tree, 313 rice accessions can be roughly clustered into 3 major groups (Fig. 1A, indicated as I, II and III).

The 313 rice accessions were inoculated with isolate *CI-4* from the *Chinese Xoo race 4*, the dominant race in South China, at the booting stage. Most of the 313 rice accessions showed highly susceptible to *Xoo*, with percent lesion length (lesion length/leaf length) more than 50% (Fig. 1B and Table S1). The modern cultivar “Xinbaoai” (No. L56) showed the strongest resistance, with percent lesion length of 0.46% (Table S1). The average percent lesion length of landraces was significantly longer than that of modern cultivars (Fig. 1C).

### Mapping of QTLs for Bacterial Blight Resistance by GWAS

**Fig. 2:**
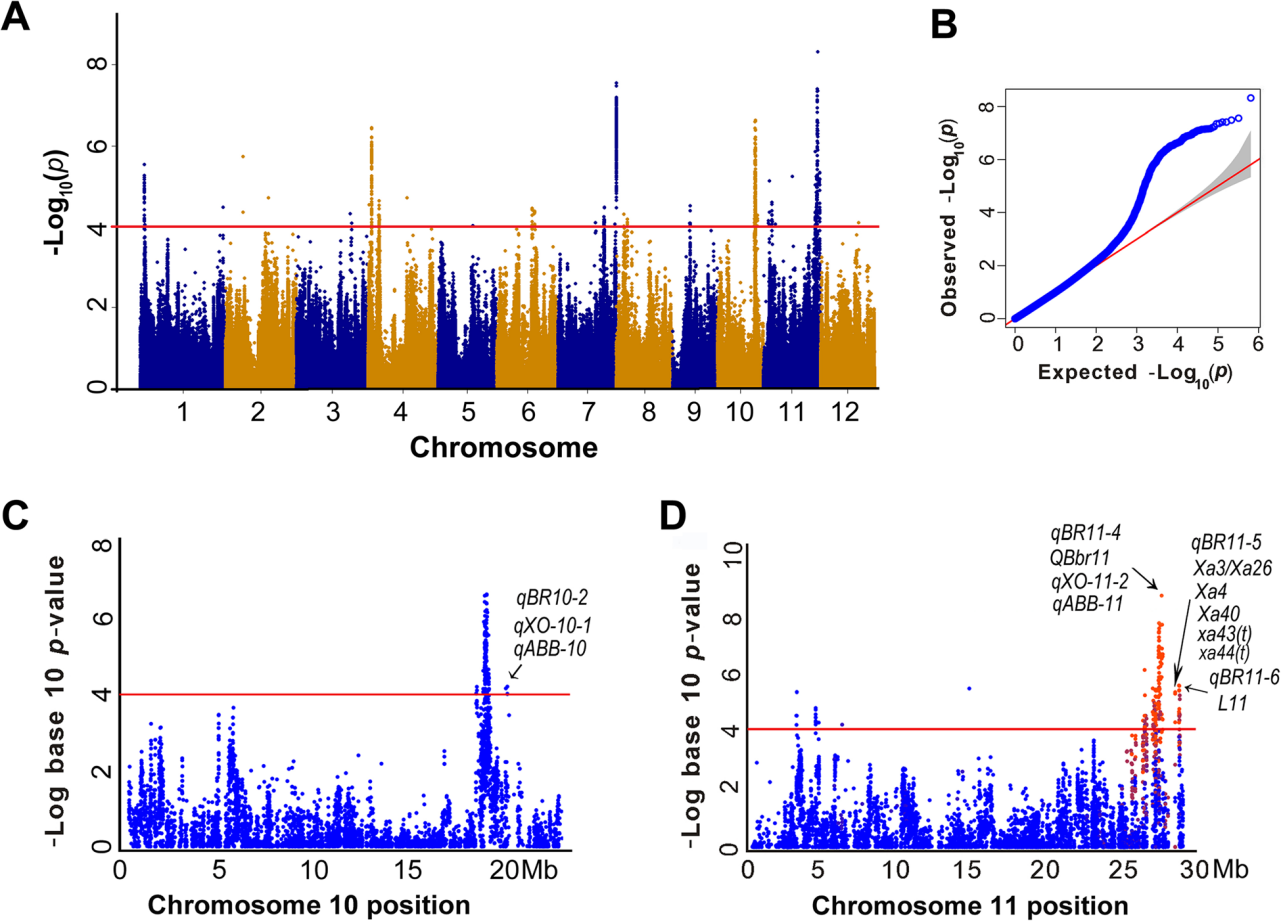
GWAS analysis for bacterial blight resistance in 313 rice accessions. **A** Manhattan plots of bacterial blight resistance in 12 chromosomes. **B** QQ-plot for GWAS of bacterial blight resistance. **C** Co-localization of *qXO-10-1* and *qABB-10* with *qBBR10–2*. **D** Co-localization of *QBbr-11*, *qXO-11-2* and *qABB-11* with *qBBR11–4*; *Xa3/Xa26*, *Xa4*, *Xa40*, *Xa43(t)* and *Xa44(t)* with *qBBR11–5*; *L11* with *qBBR11–6*. The red line indicates the significance threshold set at *P* = 1.0 × 10^− 4^

Based on the criteria of having less than 15% missing data and minor allele frequency (MAF) larger than 5% in the population, 643,841 SNPs were selected for GWAS analysis. Totally, 17 QTLs with 716 SNPs were significantly associated with bacterial blight resistance in the 313 rice accessions (Table 1, Fig. 2A and Table S2). These QTLs (designate as *qBBR* hereafter) were distributed on chromosomes 1, 4, 6, 7, 8, 9, 10 and 11, respectively (Table 1 and Fig. 2A). Compared with the previous reports, four QTLs (*qBBR10–2*, *qBBR11–4*, *qBBR11–5*, *qBBR11–6*) identified in this study were co-localized with the previously reported QTLs or *Xa* genes as shown in Table 1, Fig. 2C and D. The other 13 QTLs were newly identified in the present study.

**Table 1:** QTLs associated with rice bacterial blight resistance identified in this study

QTLs	Chromosome	Linked SNP position^a^	Major/ Minor allele	*P*-value	Phenotypic variation explained (%)	Overlapping locus/gene	Reference
*qBBR1*	1	1,936,376	A/G	2.84838E-06	4.03		
*qBBR4–1*	4	1,845,370	A/G	3.51716E-07	4.81		
*qBBR4–2*	4	5,493,782	G/A	2.26586E-05	3.28		
*qBBR6–1*	6	18,070,061	A/C	3.53715E-05	3.12		
*qBBR6–2*	6	19,329,090	T/C	4.16516E-05	3.06		
*qBBR7–1*	7	23,433,761	G/A	3.2846E-05	3.14		
*qBBR7–2*	7	29,527,042	A/G	2.74836E-08	5.78		
*qBBR8*	8	5,432,150	C/T	6.56414E-05	2.90		
*qBBR9*	9	8,986,663	G/A	3.03815E-05	3.17		
*qBBR10–1*	10	19,158,528	A/G	2.32191E-07	4.96		
*qBBR10–2*	10	20,261,868	T/C	5.27067E-05	2.97	*qXO-10-1, qABB-10*	Gustave et al. 2016; Bossa-Castro et al. 2018
*qBBR11–1*	11	3,064,808	A/T	4.41216E-05	3.04		
*qBBR11–2*	11	4,364,433	A/G	2.46251E-05	3.25		
*qBBR11–3*	11	26,432,751	C/A	1.37316E-06	4.30		
*qBBR11–4*	11	27,573,275	C/A	4.72312E-09	6.46	*QBbr11, qXO-11-2, qABB-11*	Zhang et al. 2015; Gustave et al. 2016; Bossa-Castro et al. 2018
*qBBR11–5*	11	28,484,065	G/A	7.47E-06	3.68	*Xa3/Xa26, Xa4, Xa40, xa43(t)*,*xa44(t)*	Sun et al. 2003; Cao et al. 2007; Kim et al. 2015; Kim 2018; Kim and Reinke 2019
*qBBR11–6*	11	28,759,500	C/T	4.64E-06	3.85	*L11*	Zhang et al. 2017

^a^The the most significant SNP position (bp) within QTL

### Candidate Gene Analysis of *qBBR11–4*

**Fig. 3:**
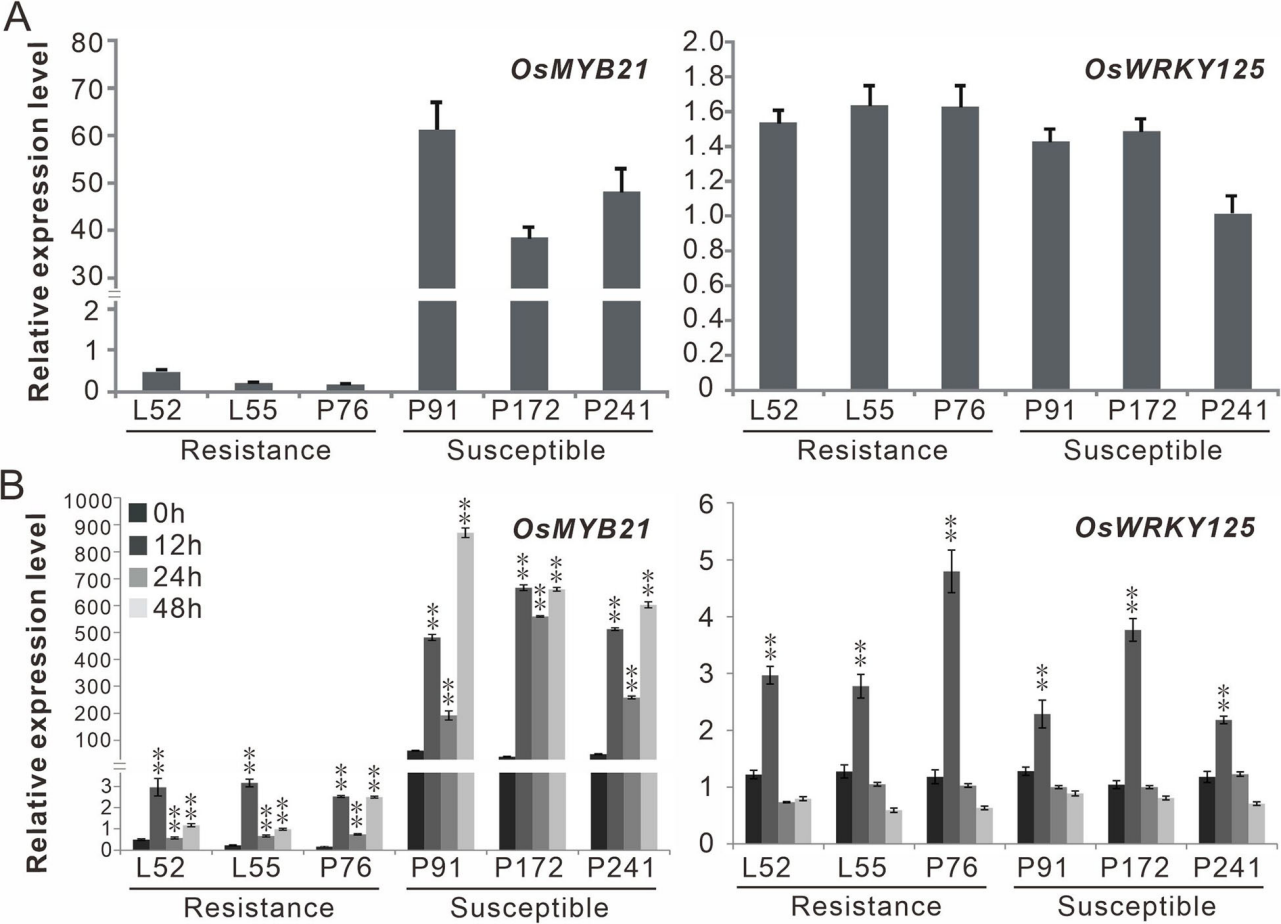
Transcription analysis of *OsMYB21* and *OsWRKY125* in the resistant and susceptible rice accessions. **A** The background expression of *OsMYB21* and *OsWRKY125* in different rice accessions. **B** The expression levels of *OsMYB21* and *OsWRKY125* in different rice accessions before (0 h) and after (6 h, 12 h and 24 h) *Xoo* inoculation. L52, L55 and P76 are the resistant accessions, while P91, P172 and P241 are the susceptible accessions. The values are means ± SDs of three biological replicates and the asterisks represent significant differences relative to 0 h treatment at **P < 0.01 by *t*-test. “Relative expression” indicates relative expression to the reference gene EF1α

Among the 17 QTLs identified in the present study, *qBBR11–4* explained the largest phenotypic variation in the population (Table 1). Moreover, *qBBR11–4* was co-localized with the previously reported QTLs (*QBbr11*, *qABB-11* and *qXO-11-2*), which were detected to be resistant to the strains from different pathotypes in Africa and Asia (Zhang et al. 2015; Djedatin et al. 2016; Bossa-Castro et al. 2018), suggesting its broad spectrum resistance to BB and potential value in rice breeding. Therefore, we further conducted candidate gene analysis of *qBBR11–4*. Because the rice linkage disequilibrium block is about 100–200 kb (Wang et al. 2020), a 400-kb region (27.37–27.77 Mb, 200 kb up and down stream of the most significant SNP within *qBBR11–4*) was used for candidate gene analysis. According to the Rice Annotation Project (RAP) (Kawahara et al. 2013), the region contains 28 functionally annotated genes excluding the retrotransposons. Referring to the gene function annotation, gene ontology classification and RNA-seq FPKM expression data shown in RAP, we lastly selected 11 potential candidate genes for transcription analysis (Table S3). Based on the diseased phenotypes and haplotype analysis of *qBBR11–4*, 3 rice accessions with *Xoo*-resistance haplotype and *Xoo*-resistance phenotype, and 3 rice accessions with *Xoo*-susceptible haplotype and *Xoo*-susceptible phenotype were selected for gene differential expression analysis. The expression levels of 11 potential candidate genes in the 6 rice accession were evaluated both befor and after pathogen inoculation. The real-time PCR analyses were conducted at 0 h, 12 h, 24 h and 48 h, respectively. The results showed that only two genes (*LOC_Os11g45740,* i.e. *OsMYB21* and *LOC_Os11g45750,* i.e. *OsWRKY125*) displayed significantly induced expression pattern after pathogen infection in both *Xoo*-resistance and *Xoo*-susceptible rice accessions (Fig. 3). Five genes showed inconsistent differential expression between the resistant and susceptible rice accessions (Figure S1), and the expression of the left four genes (*LOC_Os11g45060*, *LOC_Os11g45924*, *LOC_Os11g45920* and *LOC_Os11g45330*) were not detected in these accessions (Table S4). The transcription of *OsMYB21* was remarkably activated at all time points after pathogen inoculation, whereas the transcription of *OsWRKY125* was only increased at 12 h after *Xoo* infection (Fig. 3). Moreover, we also discovered that *OsMYB21* showed significantly lower transcript levels in *Xoo*-resistant accessions than in *Xoo*-susceptible accessions while *OsWRKY125* displayed no obvious expression difference between the two sets of varieties with contrast disease resistance (Fig. 3). We found that a 2-bp insertion in the TC-rich repeats at 154 bp before initiator codon in the promoter regions of *OsMYB21* in the *Xoo*-susceptible accessions compared with the *Xoo*-resistant accessions (Figure S2). These results together suggest that *OsMYB21* could be the candidate gene of *qBBR11–4*.

### Functional Validation of *OsMYB21*

**Fig. 4:**
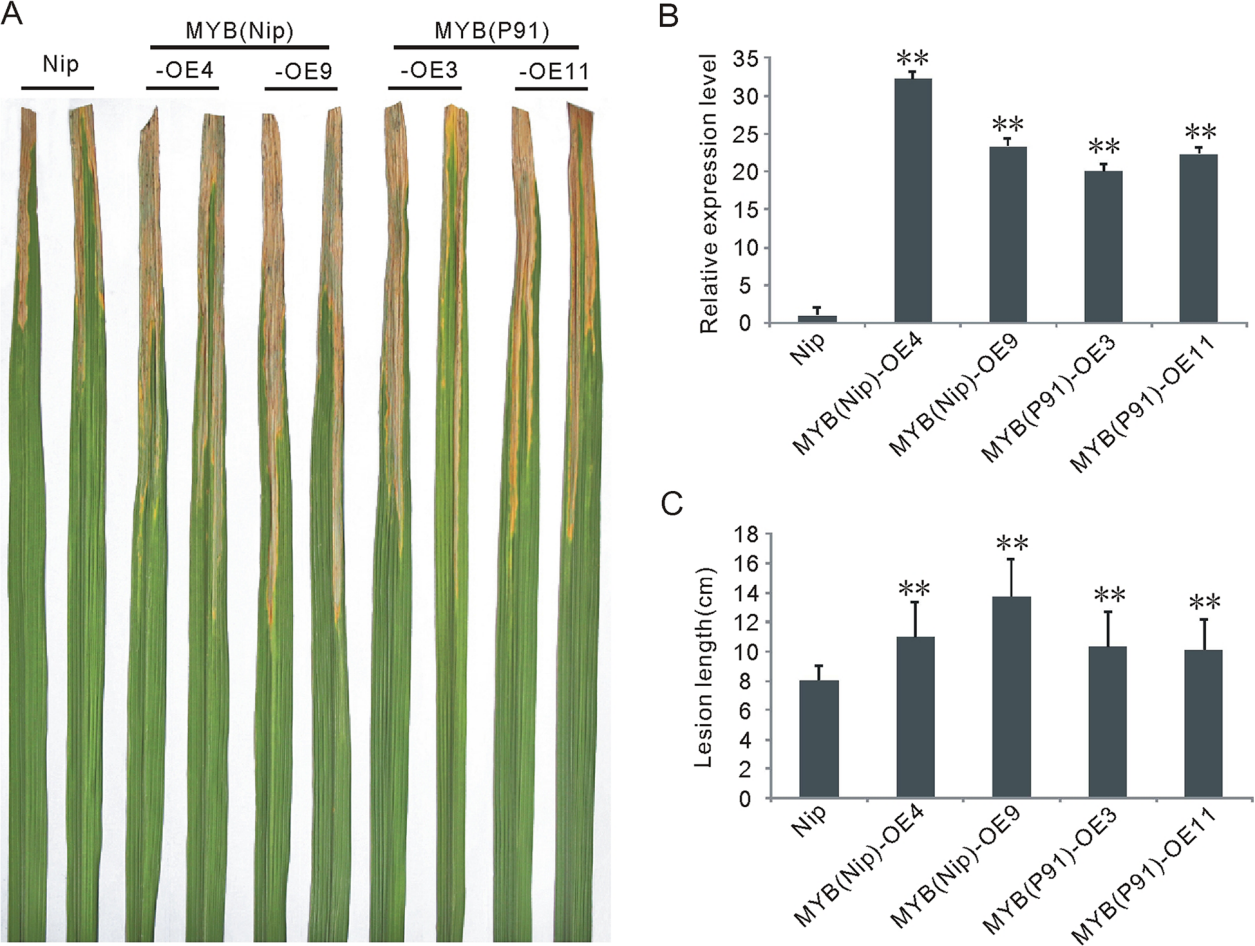
Functional validation of *OsMYB21*. **A** The phenotypes of wild-type *Nipponbare* and *OsMYB21* overexpressing plants after *Xoo* inoculation. MYB21(P91)-OE indicates the transgenic lines overexpressing the genotype “P91” of *OsMYB21*, and MYB21(Nip)-OE indicates the transgenic lines overexpressing the genotype “*Nipponbare*” of *OsMYB21*. **B** Relative expression levels of *OsMYB21* in the wild-type *Nipponbare* and transgenic plants. The values are means ± SDs of three biological replicates. **C** Relative lesion length in the wild-type *Nipponbare* and transgenic plants after *Xoo* inoculation. The values are means ± SDs of at least twenty leaves. Asterisks represent significant differences relative to 0 h treatment at **P < 0.01 by *t*-test

To confirm the function of *OsMYB21* in bacterial blight resistance, we firstly compared the protein sequences between *Nipponbare, Xoo*-resistance and *Xoo*-susceptible accessions used in transcription analysis, and found no consistent difference (Figure S3). Transgenic plants constitutively overexpressing *OsMYB21* (MYB21OE) were produced in *Nipponbare* which are susceptible to *Xoo CI-4*. Twenty-one transgenic lines overexpressing the genotype “P91” [MYB21(P91)-OE] and 24 transgenic lines overexpressing the genotype “*Nipponbare*” [MYB21(Nip)-OE] were obtained, respectively, and they all showed no significant difference in the non-target traits when compared to the wild-type plants (data not shown). Two independent homozygous lines of each genotype were selected for further evaluation. Gene transcriptional analysis revealed that the expression of *OsMYB21* was significantly increased in these transgenic plants (Fig. 4). The MYB21OE plants of two genotypes all showed remarkably increased susceptibility to bacterial blight, with lesion lengths ranging from 10.12 to 13.73 cm, compared to 8.0 cm for the wild-type *Nipponbare* plants (*p*<0.01) (Fig. 4).

### The Relationship between *OsMYB21* and Pathogenesis-Related Genes in Regulating Disease Resistance in Rice

**Fig. 5:**
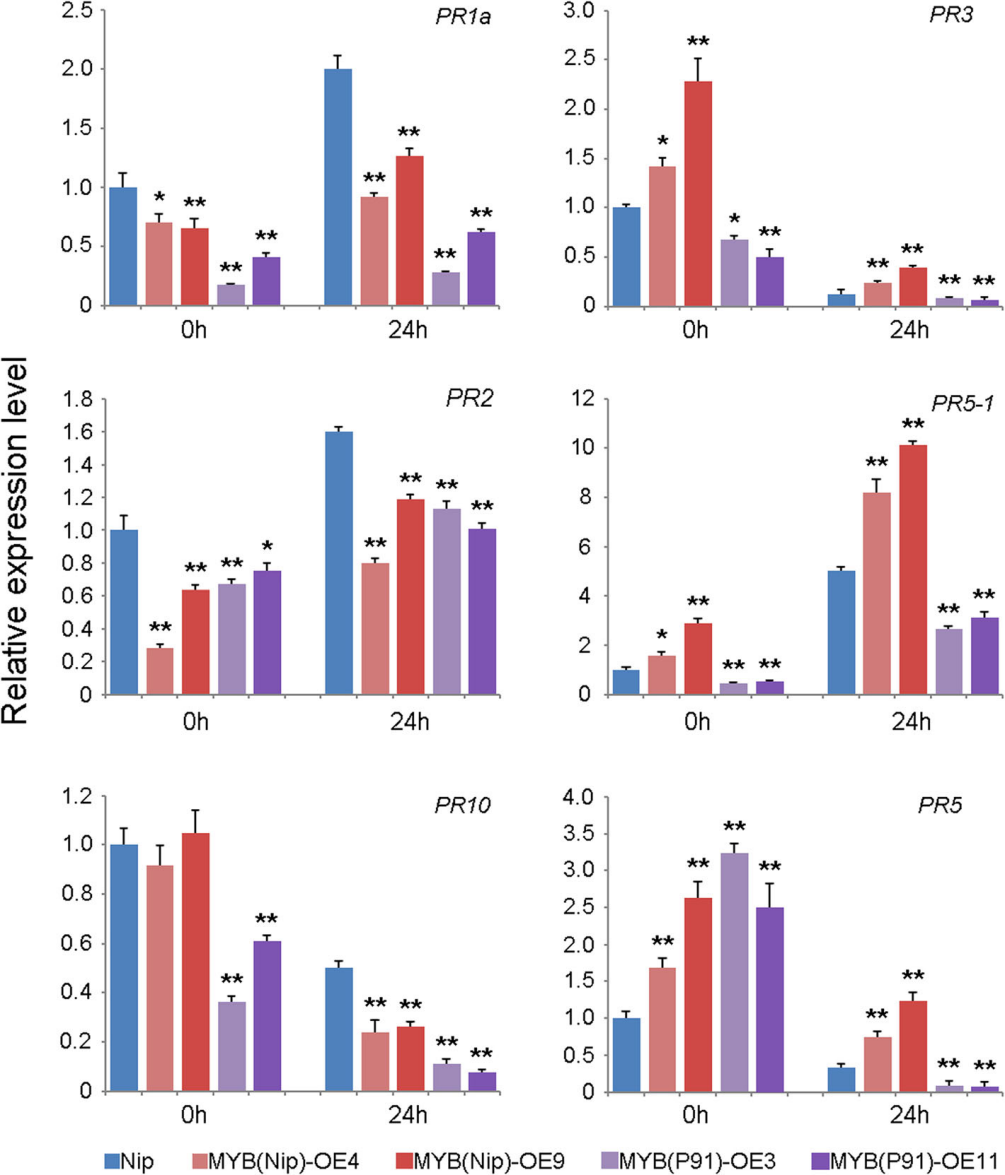
The expression levels of six *PR* genes in the wild-type *Nipponbare* and transgenic plants before (0 h) and after (24 h) *Xoo* inoculation. The values are means ± SDs of three biological replicates and the asterisks represent significant differences relative to wild-type plants at **P < 0.01 and *P < 0.05 by t-*t*est. The transcript level of *Nipponbare* was set to “1” at 0 h treatment. “Relative expression” indicates relative expression to the reference gene EF1α

Previous study has reported that three MYB transcription factors mediate disease resistance through regulation of *PR* genes in wheat (Zhang et al. 2012; Liu et al. 2013; Shan et al. 2016). To determine if *OsMYB21* mediated disease susceptibility was through regulating the expression of *PR* genes, the transcription levels of six *PR* genes were analyzed in the wild-type *Nipponbare* and four MYB21OE plants both before and after *Xoo* inoculation. The results showed that pathogen infection strongly induced the expression of *PR1a*, *PR2* and *PR5–1* but reduced the expression of *PR3*, *PR5* and *PR10* in both the wild-type and MYB21OE plants before and after *Xoo* inoculation. The transcription levels of *PR1a*, *PR2* and *PR10* were significantly down-regulated in MYB21OE plants compared with the wild-type plants before and after pathogen inoculation (Fig. 5).

## Discussion

### The *qBBR11–4* Has a Great Potential Value in Rice Breeding

In the present study, the evaluation of BB resistance of the core rice panel from South China showed that among the 313 accessions, only 5 accessions were resistant (disease scale lower than 3 or lesion area less than 12%) to *CI-4,* a BB isolate from the dominant race 4 in South China (Table S1), suggesting the difficulty in rice breeding for BB resistance. In the past decades, rice breeding for BB resistance largely relies on *R* gene resistance. This type of disease resistance is easily overcome due to the diversity and variability of the pathogen. The short-lived span of BB resistance for most of rice cultivars has been a major issue in rice production. Although deployment of the non-race specific quantitative resistance has been proposed as a good strategy to achieve durable resistance, this strategy has not been successfully carried out in rice breeding due to the polygenic nature and lack of knowledge on the functional genes underlying the QTLs.

To identify the genes associated with quantitative BB resistance, GWAS of BB resistance was performed using a diverse rice core collection from South China in this study. In total, 17 QTLs associated with BB resistance were identified. Among the 17 QTLs, *qBBR11–4* explained the largest variations of BB resistance in the population (Table 1). It is noteworthy that *qBBR11–4* overlaps with the previously identified QTLs for broad-spectrum BB resistance, *qXO-11-2, qABB-11* and *QBbr11* (Zhang et al. 2015; Djedatin et al. 2016; Bossa-Castro et al. 2018). The *qXO-11-2* was detected to be resistant to five African *Xoo* strains from different pathotypes (Bossa-Castro et al. 2018) while *qABB-11* was detected to be resistant to four African strains and the Philippine isolate PXO61 (Djedatin et al. 2016), and *QBbr11* was identified to be resistant to three isolates representing Pathotype II, Pathotype IV and Pathotype V, respectively (Zhang et al. 2015). These results together suggest that the *qBBR11–4* identified in the present study could be a broad-spectrum BB resistant QTL. Its large phenotypic effect and broad-spectrum BB resistance make *qBBR11–4* have a great potential value in rice breeding.

### *OsMYB21* Can Be a Promising Target for Improvement of BB Resistance in Rice

To identify the gene underlying *qBBR11–4*, a 400-kb region was chosen for gene annotation referring to the previous reports (Kawahara et al. 2013; Zhao et al. 2018). After filtering the retrotransposons, non-expressed genes and differential expression analysis of the genes within *qBBR11–4* using two sets of rice accessions with contrast BB resistance before and after *Xoo* inoculation, *OsMYB21* was identified as the only candidate gene for *qBBR11–4* (Fig. 3, Figure S1 and Table S4). Based on DNA re-sequencing, a 2-bp variation in the TC-rich repeats of the promoter was found (Figure S2). TC-rich repeats have been detected in the promoter region of many plant disease-resistance genes (Germain et al. 2012). Therefore, the 2-bp difference in the promoter may result in different *OsMYB21* expression profiles in the resistant cultivars and susceptible cultivars. The previous study reported that *OsMYB21* carried the binding target site of TAL effector of XOO2127_MAFF (Grau et al. 2013), further suggesting the possible role of *OsMYB21* in bacterial blight resistance. The transgenic plants overexpressing *OsMYB21* exhibited decreased bacterial blight resistance as manifested by the longer lesions compared with wild-type *Nipponbare* plants (Fig. 4). Thus, our results suggest that *OsMYB21* is the causal gene of *qBBR11–4* and negatively regulates BB resistance in rice. The negative role of *OsMYB21* implies that this gene can be a promising target for improvement of BB resistance in rice by means of gene editing method. It is noteworthy that the induction of *OsMYB21* by the BB isolate *CI-4* and the bacterial blight susceptibility mediated by *OsMYB21* might be related to the TAL effector of *CI-4*. It will be particularly interesting to determine if any TAL effector targets overlap the 2-bp deletion found in *Xoo*-resistant accessions.

As one of the largest families of transcription factors, many MYB genes have been demonstrated as important regulators in plant responses to both biotic and abiotic stresses (Mengiste et al. 2003; Yang et al. 2012; Zhang et al. 2012; Zhu et al. 2015). In rice, 233 MYB genes have been identified (Smita et al. 2015). Nevertheless, only *MYB30*, *MYB55*, and *MYB110* have been validated to positively regulate rice bacterial blight resistance by activating the hydroxycinnamic acids synthesis pathway (Kishi-Kaboshi et al. 2018). Our findings in the present study enrich our knowledge about the functions of MYB genes in disease resistance. Additionally, *OsMYB21* has been shown down-regulated over *ustilaginoidea virens*, which causes rice false smut (Wei et al. 2020). In *Arabidopsis*, glutathione could activate the heat shock proteins through the transcription factor MYB21 (Kumar and Chattopadhyay 2018). In *Pyrus betulaefolia*, *MYB21* plays a positive role in drought tolerance (Li et al. 2017). Therefore, we speculate that *OsMYB21* may have important roles in pathways for other biotic and abiotic stresses than just being a negative regulator to bacterial blight in rice.

### The Novel QTLs Provides a New Source for Molecular Rice Breeding and Cloning of Genes Associated with BB Resistance in Rice

Among the 17 QTLs for BB resistance identified in this study, 4 QTLs (*qBBR10–2*, *qBBR11–4*, *qBBR11–5* and *qBBR11–6*) were co-localized with the previously identified *R* genes or QTLs for BB resistance and the other 13 QTLs were newly identified in the present study (Table 1), indicating the reliability of our GWAS analysis and the diversity of rice accessions used in this study. We have listed the predicted genes of the putative intervals of the 13 QTLs and their potential candidate genes (Table S3). We discovered that the lead SNPs of two QTLs (*qBBR4–1* and *qBBR7–1*) were located in the protein coding regions of genes encoding YT521-B-like protein family protein and AP2/EREBP transcription factor BABY BOOM, respectively (Table S3). AP2/EREBPs belong to a superfamily of plant-specific transcription factors that containing an AP2 domain (Li et al. 2016). According to the previous reports, many members of AP2/EREBP family have been validated to positively modulate plant disease resistance by regulating the transcription of defense-related genes (Park et al. 2001; Guo et al. 2004; Li et al. 2011; Lu et al. 2013; Giri et al. 2014). For instance, overexpression of OPBP1, an AP2/EREBP-like transcription factor of tobacco, enhances disease resistance in both tobacco and rice plants (Guo et al. 2004; Chen and Guo 2008). Thus, the BABY BOOM gene might also play a role in mediating rice against *Xoo* infection. Expect for the BABY BOOM, we also find three wall-associated protein kinases (WAK1, WAK2 and WAK29) among the candidate genes underlying *qBBR1* and *qBBR4–1* (Table S3)*.* Generally, WAK, which has the ability to link plasma membrane to cell wall matrix, is one of the most likely target genes functioning in plant defense response by directly signaling cellular events through their cytoplasmic kinase domain (Li et al. 2009). Recently, a new BB resistance gene, *Xa40*, was identified by using graphical mapping, and examination of the candidate genes showed that only *WAK3* transcription levels displayed significant differences (Kim et al. 2015). Also, *WAK25*, *WAK14*, *WAK91* and *WAK92* were reported to positively regulate rice blast or *Xoo* resistance, while *WAK112d* was shown to negatively mediate rice blast resistance (Delteil et al. 2016; Harkenrider et al. 2016). Therefore, we deduced that the three WAK genes could be the candidate genes of *qBBR1* and *qBBR4–1*. Further studies are needed to confirm their functions of these candidate genes in *Xoo* resistance through gain or loss-of function analysis. The identification of 13 novel QTLs for BB resistance provides a new source for molecular rice breeding and cloning of genes associated with BB resistance in rice.

## Conclusion

In the present study, large BB resistance variations within 313 rice accessions from South China were observed. Among the 17 QTLs identified in this study, 4 QTLs were co-localized with the previously reported QTLs or *Xa* genes. The *qBBR11–4* on chromosome 11 explained the largest phenotypic variation and was co-localized with the previously identified QTLs for BB and bacterial leaf streak resistance against diverse strains in three studies, suggesting its broad-spectrum resistance and potential value in rice breeding. Since overexpressing *OsMYB21* decreased resistance to bacterial blight, *OsMYB21* functions as a negative regulator in bacterial blight resistance in rice, providing a promising target in rice improvement of BB resistance by means of gene editing, specifically by introducing the 2-bp difference in the promoter of *OsMYB21*. In addition, the 13 novel QTLs for BB resistance were detected in this study and the potential candidate genes for these novel QTLs were analyzed, providing a new source for cloning of genes associated with BB resistance and molecular breeding in rice.

## Materials and Methods

### Plant Materials and Pathogen

The 313 rice core germplasms (*indica* rice) composed of 255 landraces and 58 modern cultivars were used for GWAS analysis (Table S1). They were collected in South China by the Rice Research Institute, Guangdong Academy of Agricultural Sciences. The 313 rice accessions represent 90% of the diversity of original collection in term of 26 traits (unpublished data). Rice cultivar *Nipponbare* (*ssp. japonica*) was used for the transgenic analysis and *Chinese Xoo race 4* (*CI-4*) was used for evaluation of bacterial blight resistance.

### Sequencing, SNP Calling and Phylogenetic Analysis

All 313 accessions were sequenced by Illumina Hiseq2000 platform. Raw sequencing reads were mapped to rice reference genome sequence version of MSU V7.0 (Kawahara et al. 2013) by Bowtie2 (Langmead and Salzberg 2013) and SNP were called and filtered according to GATK3.8 best practices pipeline (McKenna et al. 2010). SNP were then further filtered by the criteria of having less than 15% missing data and minor allele frequency (MAF) > 0.05 by TASSEL 5.0 (Bradbury et al. 2007). Finally 643,841 SNP were identified for further phylogenetic and GWAS analysis. Maximum-Likelihood (ML) phylogenetic tree were conducted by MEGA 7.0 (Kumar et al. 2016) using all 643,841 SNP above. The program operating parameters were set as follows: a Tamura-Nei model with 1000 bootstrap repetitions, accompanied by uniform rates, and partial deletion of gaps/missing data.

### Pathogen Inoculation and Disease Severity Evaluation

For GWAS analysis, 313 rice accessions were planted in the paddy field at the experimental base of Guangdong Academy of Agricultural Sciences in Guangdong, China, in the second cropping season in 2017. The experiments were arranged in a randomized complete block design with two replicates. The field management including irrigation, fertilization, and disease and pest control, followed the conventional practice in rice production. More than 24 leaves from 8 individual plants in the middle of each line were inoculated with *CI-4* at the booting stage using the leaf-clipping method (Kauffman et al. 1973). Disease was assessed by measuring the percent lesion length (lesion length/leaf length) at 2 weeks after inoculation. Since large variations in leaf length were observed among the 313 accessions used in this study, we used the “percent lesion length” for disease evaluation of the rice accession in order to better reflect the difference in disease severity among these diverse rice accessions.

For transcription analysis and phenotypic evaluation of transgenic plants, plants were grown in soil in the greenhouse and inoculated with *CI-4* at the booting stage by the leaf-clipping method. Disease severity was assessed by measuring the lesion length. RNA samples for transcription analysis were collected at 0 h, 12 h, 24 h and 48 h after pathogen inoculation. The experiments were repeated twice.

### GWAS Analysis, QTL Delimitation and Identification of Candidate Gene

GWAS analysis, QTL delimitation and identification of candidate gene were performed using the same method as described in our previous study (Zhao et al. 2018). Briefly, all 643,841 SNPs and GAPIT version 2 were used for GWAS analysis (Tang et al. 2016). GWAS was conducted using the mix liner model with kinship matrix, and the principle component (PC) was set to 2 in GAPIT. Manhattan and QQ plots were produced using R package qqman (Turner 2014). A QTL was declared if a region has two or more than two significant SNPs within a 200 kb interval. The candidate genes were searched from 200 kb upstream and downstream of the most significant SNP in each QTL.

### Real-Time PCR Analysis

Real-time PCR was conducted using the same method as described by Liu et al. (Liu et al. 2016) with minor modifications. Total RNA was extracted from rice leaf tissues with Eastep® Super Total RNA Extraction Kit (Promega Biotech Co., Ltd., USA). The PCR analysis was performed using the BioRad CFX 96 system. All experiments were repeated thrice and the gene-specific primers used in this study were listed in Table S5.

### Vector Construction and Rice Transformation

The coding sequences of *OsMYB21* were amplified from rice varieties P91 and *Nipponbare*, respectively, using OsMYB21-OE-F/R primers (Table S5), and were then sub-cloned into the pOx overexpressing vector under control of the ubiquitin promoter. The constructed plasmids were re-checked by sequencing and transferred into *Nipponbare* via *Agrobacterium tumefaciens* EHA105 by an Agrobacterium-mediated genetic transformation approach.

## Supplementary Information

**Additional file 1: Table S1.** Information on the 313 diverse genotypes used in this study.

**Additional file 2: Table S2.** 716 SNPs were significantly associated with bacterial blight resistance in the 313 rice accessions.

**Additional file 3: Table S3.** Locus overlapping or flanking the SNP identified by GWAS.

**Additional file 4: Table S4.** Expression detection of 11 potential genes before and after bacterial inoculation.

**Additional file 5: Table S5.** Primers used for quantitative RT-PCR and vector construction.

**Additional file 6: Figure S1.** The expression levels of five candidate genes in different rice accessions before (0 h) and after (6 h, 12 h and 24 h) *Xoo* inoculation.

**Additional file 7: Figure S2.** Sequence variations in the promoter region of *OsMYB21* between *Xoo*-resistant and *Xoo*-susceptible accessions.

**Additional file 8: Figure S3.** Alignment of protein sequences between *Nipponbare*, *Xoo*-resistance and *Xoo*-susceptible accessions.

## Data Availability

The datasets supporting the conclusions of this article are provided within the article and its additional files.

## Abbreviations

BB: Bacterial blight
GWAS: Genome-wide association study
BLS: Bacterial leaf streak
*Xoo*: *Xanthomonas oryzae* pv. *oryzae*
QTL: Quantitative trait loci
PR: Pathogenesis-related
SNP: Single-nucleotide polymorphism
MAF: Minor allele frequency
