# Supplementary figures and images for: Genome-Wide Association Mapping and Gene Expression Analysis Reveal the Negative Role of OsMYB21 in Regulating Bacterial Blight Resistance in Rice
Source: Rice (N Y). 2021 Jun 29;14:58. doi: 10.1186/s12284-021-00501-z (PMC8241976; doi:10.1186/s12284-021-00501-z)

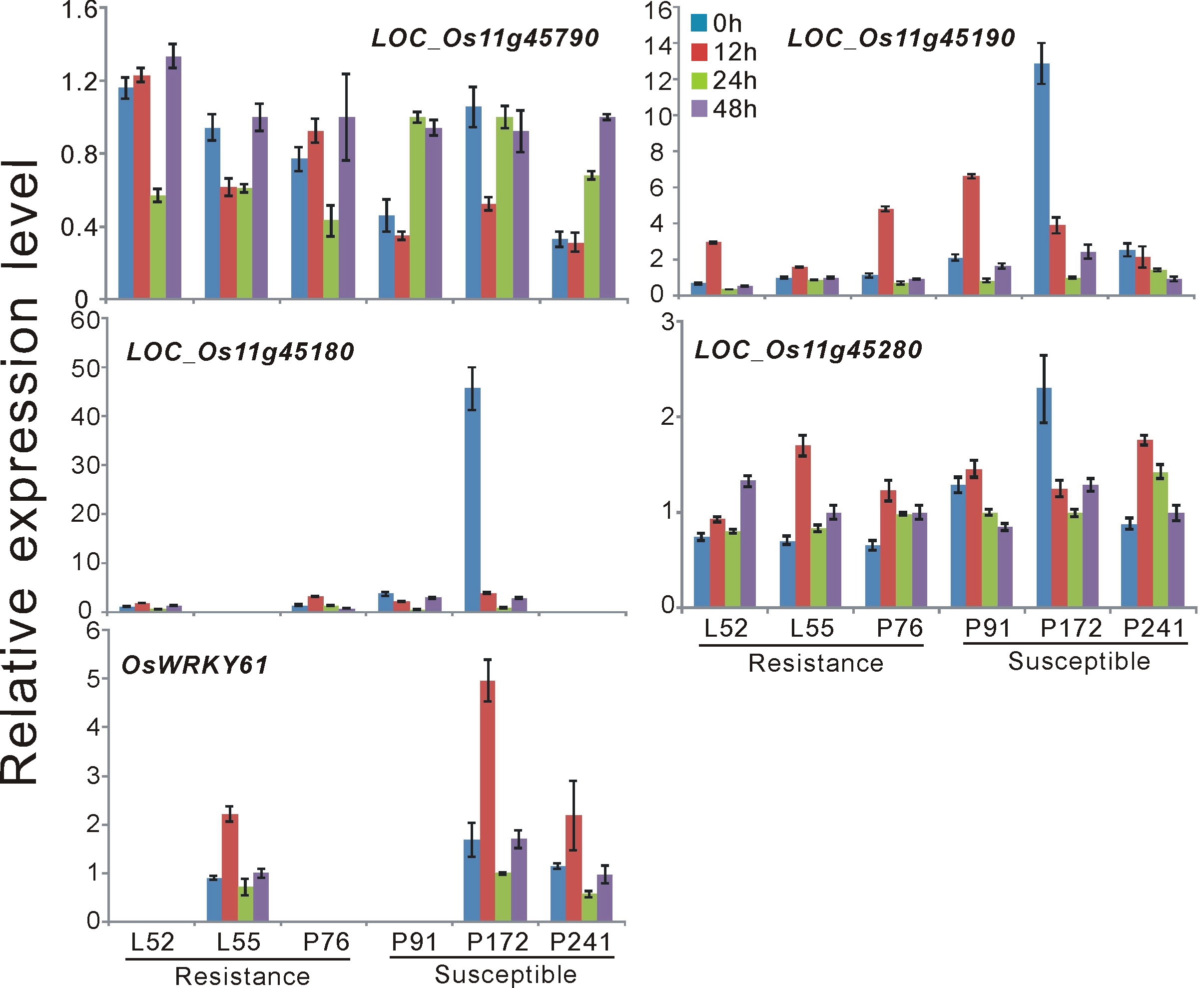

Supplement: Supplementary file 6 — Additional file 6: Figure S1. The expression levels of five candidate genes in different rice accessions before (0 h) and after (6 h, 12 h and 24 h) Xoo inoculation. [file 12284_2021_501_MOESM6_ESM.jpg]

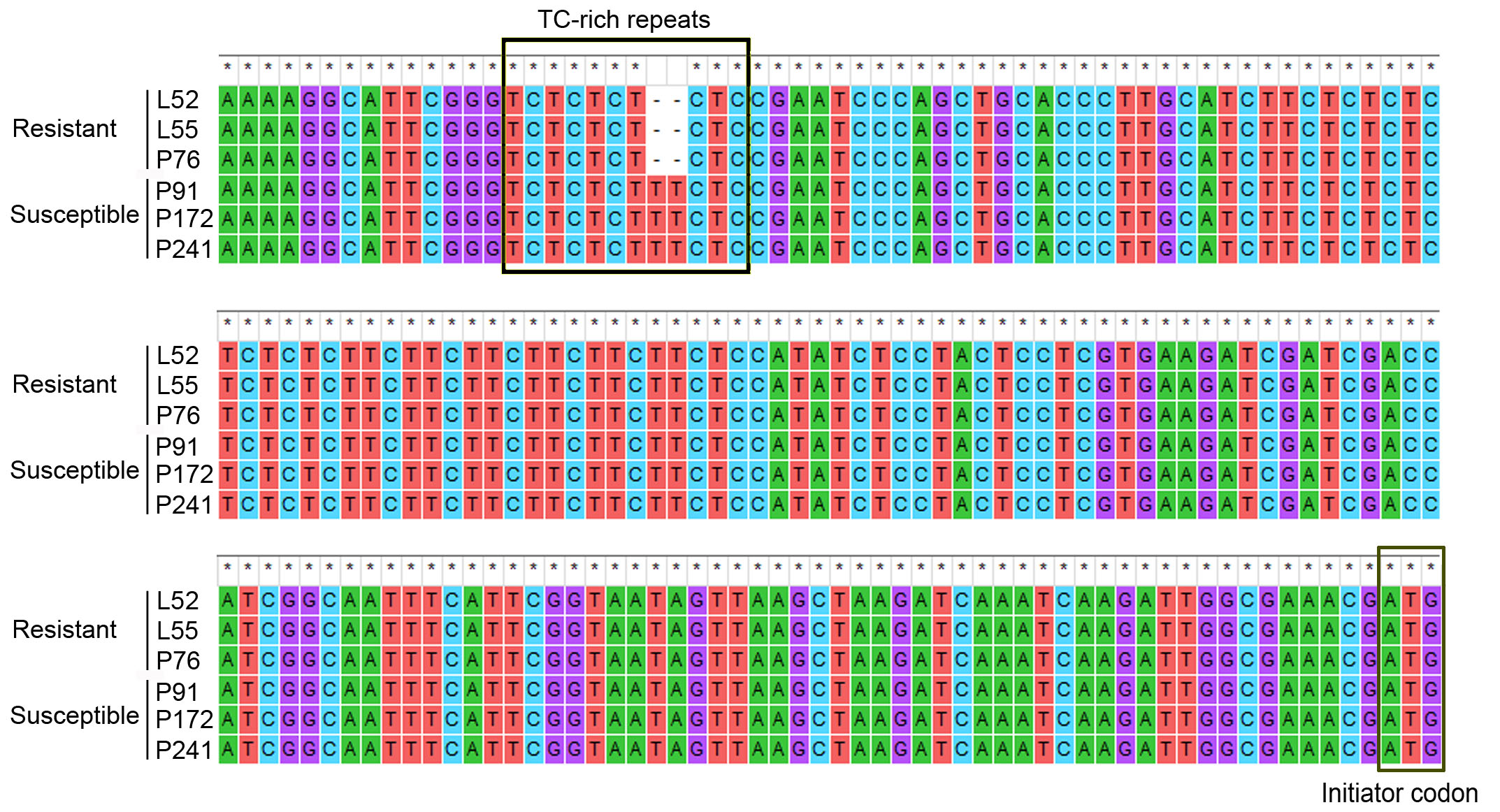

Supplement: Supplementary file 7 — Additional file 7: Figure S2. Sequence variations in the promoter region of OsMYB21 between Xoo-resistant and Xoo-susceptible accessions. [file 12284_2021_501_MOESM7_ESM.jpg]

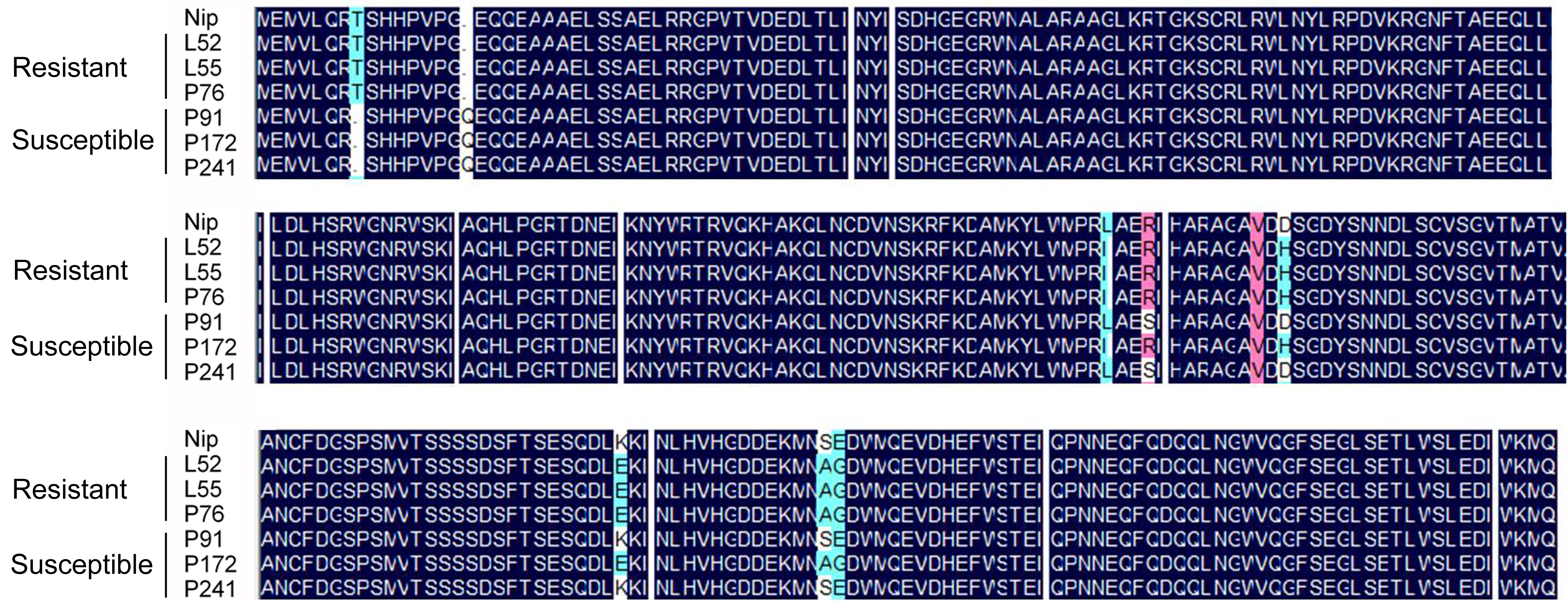

Supplement: Supplementary file 8 — Additional file 8: Figure S3. Alignment of protein sequences between Nipponbare, Xoo-resistance and Xoo-susceptible accessions. [file 12284_2021_501_MOESM8_ESM.jpg]
